# Clinical characteristics and prognostic significance of EBER positivity in diffuse large B-cell lymphoma: A meta-analysis

**DOI:** 10.1371/journal.pone.0199398

**Published:** 2018-06-19

**Authors:** Xiaojuan Gao, Jia Li, Yaqi Wang, Shuai Liu, Baohong Yue

**Affiliations:** 1 Department of Laboratory Medicine, the First Affiliated Hospital of Zhengzhou University, Zhengzhou, P. R. China; 2 Faculty of Laboratory Medicine, Zhengzhou University, Zhengzhou, P. R. China; 3 Key Laboratory Medicine of Henan Province, Faculty of Laboratory Medicine of Zhengzhou University, Zhengzhou, P. R. China; 4 Open Laboratory, Henan Province Key Subject of Clinical Medicine, Zhengzhou, P. R. China; Fondazione IRCCS Istituto Nazionale dei Tumori, ITALY

## Abstract

Recent studies show that Epstein-Barr virus (EBV) positivity might be related to adverse prognosis in patients with diffuse large B-cell lymphoma (DLBCL), but the results are still inconclusive. We conducted this meta-analysis to define the clinical value of EBV infection in DLBCL. All potential articles in PubMed, Web of Science, Medline, and Embase were retrieved. Using the random-effects or fixed-effect model, pooled hazard ratios (HRs) or relative risk (RR) with 95% confidence intervals (CIs) were used to calculate the correlation between EBER and prognosis and clinical features in DLBCL. A total of 13 qualified studies with 4111 patients were identified in our meta-analysis based on the inclusion and exclusion criteria. The overall estimates revealed that EBV-encoded small RNAs (EBER) positivity was significantly correlated with worse overall survival (HR = 2.43, 95% CI: 1.73–3.36) and progression-free survival (HR = 3.60, 95% CI: 2.07–6.26). In addition, EBER positivity was associated with age older than 60 years (RR = 1.51, 95% CI: 1.02–2.24), male sex (RR = 1.34, 95% CI: 1.05–1.71), more advanced stage (RR = 2.25, 95% CI: 1.72–2.96), high international prognostic index (RR = 2.20, 95% CI: 1.71–2.82), more than one extranodal involvement (RR = 1.69, 95% CI: 1.27–2.26), presence of B symptom (RR = 1.75, 95% CI: 1.30–2.35), non-germinal center B-cell subtype (RR = 1.35, 95% CI: 1.03–1.78), and elevated lactate dehydrogenase levels (RR = 1.30, 95% CI: 0.98–1.72). EBER positivity was correlated with worse outcomes, worse clinical course, and adverse clinicopathologic features among patients with DLBCL.

## Introduction

Diffuse large B-cell lymphoma (DLBCL) is the most common type of non-Hodgkin lymphoma, accounting for 30%–40% of all initially diagnosed cases [1]. It is an invasive lymphoma with heterogeneous histology, clinical features, surface marker expression, and clinical outcomes [2]. Only 50% of the patients with DLBCL achieve persistent remission.

The International Prognostic Index (IPI) is the most extensively used prognostic indicator in DLBCL [3]; it includes five clinical variables, namely, age, disease stage, performance status, serum lactate dehydrogenase (LDH) level, and the number of extranodal sites. However, the IPI score does not reflect the biological changes of tumor patients with DLBCL. Recently, the development of rituximab has transformed the chemotherapy regimen for aggressive lymphoma and led to remarkable progress in the outcome of DLBCL regardless of its IPI risk category [4]. In the rituximab era, the limitations of IPI score have become more prominent, and studies shown that the ability of IPI to identify risk groups has decreased [5]. In addition, considering the heterogeneous clinicopathological and genetic features of DLBCL, some reports suggested that although it is easy to implement, the IPI may not fully predict the prognosis of DLBCL [6–9]. Hence, identifying other valuable prognostic factors that can be used for the stratified treatment of DLBCL is crucial.

Epstein–Barr virus (EBV) infection is related to some lymphoid malignancies, such as Burkitt lymphoma, natural killer-cell leukemia/lymphoma, DLBCL, and a proportion of Hodgkin lymphoma cases, among others. Reports on the frequency of EBV-encoded small RNA (EBER)-positive DLBCL in different geographic regions have been substantially inconsistent, with the incidence being higher in Asian countries such as Japan and Korea (8%–9%) [10–12] than in some Western countries (1%–3%) [13, 14]. Many studies have focused on the prognostic value of EBV infection in DLBCL to determine potential prognostic markers and indicators for stratified treatment. However, the clinicopathological features and prognostic value of EBER positivity in patients with DLBCL remain controversial. Some studies showed that EBER positivity has an important prognostic value in DLBCL [10,11,15–20], whereas other studies reported opposite results [14,21–24]. Furthermore, determining prognostic factors at initial diagnosis may be helpful in providing risk-based stratification treatment for DLBCL and in identifying patients who need early intensive therapy. Thus, this meta-analysis was conducted to evaluate the prognostic value of EBER positivity in patients with DLBCL. Subgroup analyses was also conducted to further identify the association between EBER positivity and the clinical features of DLBCL. To the best of our knowledge, this is the first meta-analysis on such topic.

## Methods

### Search strategy

We conducted a systematic electronic search of PubMed, Embase, Medline, and Web of Science for potential articles published before February 31, 2018. We identified articles using the following corresponding keywords: “Epstein-Barr Virus,” “EB virus,” “EBV,” “human herpesvirus 4,” “HHV 4,” “diffuse large B cell lymphoma,” and “DLBCL.” No region limitation was adopted. Moreover, we manually checked the relevant studies to prevent omission of any research. We contacted authors and asked for additional information if the key data relevant to this meta-analysis were insufficient.

### Selection criteria and quality assessment

Studies were selected if they met the following inclusion criteria: (1) confirmed histopathological diagnosis of DLBCL cases; (2) the prognostic value of EBER positivity among patients with DLBCL was estimated; (3) sufficient survival data to calculate the hazard ratio (HR) with 95% confidence interval (CI) were provided; (4) EBER was detected via in-situ hybridization; (5) more than 30 patients were enrolled in each study. When the same patient population was described in several reports, only the most informative one was included. Case reports, editorials, reviews, letters, conference abstracts, comments, and studies published in languages other than English were excluded. Each included study was independently evaluated by two investigators using the Newcastle Ottawa Scale [25]. Moderate- to high-quality studies were defined as those with NOS scores of more than six points.

### Data extraction

Two investigators independently extracted data from qualified articles. Disagreements were settled by consulting with a third reviewer. Clinical characteristics and prognosis data were extracted from the included studies. Information collected from each eligible study included the first author’s name, study location, publication year, number of patients, sex, median follow-up, thresholds, EBV status, and outcome correlation. We selected overall survival (OS) and progression free survival (PFS) as endpoints in this meta-analysis. The HR and its 95% CI were directly extracted from the original study or indirectly estimated from the Kaplan-Meier curves using software designed by Tierney et al. [26].

### Statistical analysis

The pooled HR and the corresponding 95% CIs for OS and PFS were estimated using the random-effect model. An HR >1 indicated an unfavorable prognosis in EBER-positive DLBCL patients. In the analysis of the correlation between EBER positivity and clinicopathologic features (age, sex, disease stage, number of extranodal sites, serum LDH level, B symptom, histologic subtype, and IPI score), the relative risk (RR) was estimated via the fixed-effect model. An RR >1 suggested that EBER positivity was correlated to the parameter. Cochran’s Q test was used to assess the heterogeneity across studies, and *P*<0.10 or I^2^ statistic >50% was deemed to be statistically significant. The fixed-effects model was applied for *P*>0.10 or I^2^<50%; otherwise, the random-effects model was applied. We performed a sensitivity analysis by sequentially omitting each study to verify the stability of the results. Subgroup analyses were also performed based on the geographical setting of the study, which might be a potential source of heterogeneity. Begg’s and Egger’s tests were conducted to investigate potential publication bias. All statistical analyses were performed using Stata Version 12.0. *P*<0.05 was considered statistically significant.

## Results

### Selection and characteristics of the studies

**Fig 1:**
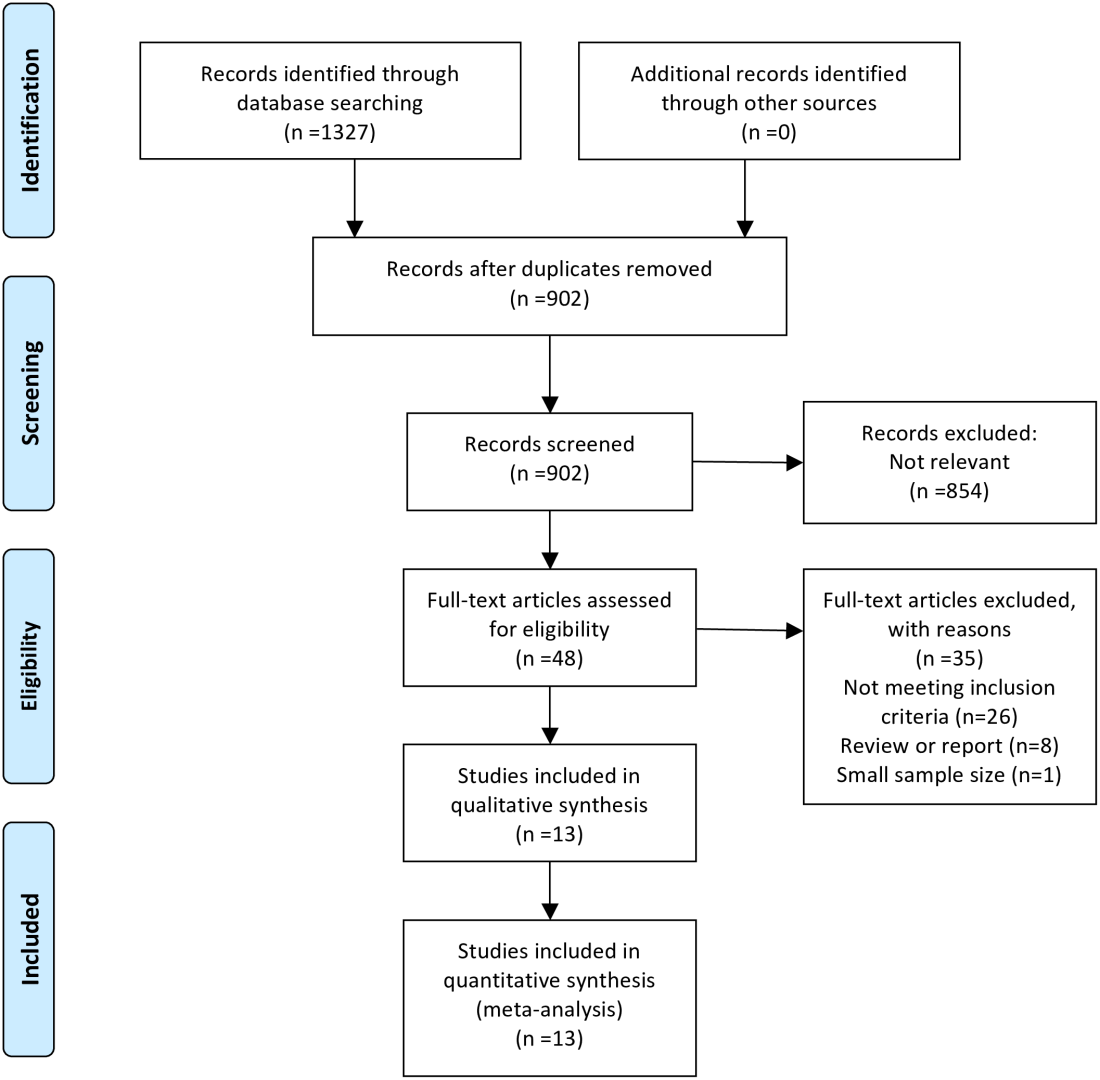
Flow diagram of the included studies.

The search strategy is shown in Fig 1. A total of 1327 studies were retrieved for detailed screening. Of these, 1279 studies were excluded due to duplication (n = 425) and irrelevance or them being another type of study other than research (n = 854). The full text of the 48 potentially qualified articles were reviewed. Finally, after excluding those with non-survival analysis data or those whose HR was not obtained, 13 articles [10,11,14–24] were included in the meta-analysis.

The baseline features of the 13 eligible studies are presented in Table 1. A total of 4111 participants (ranging from 74 to 703 for each article) were included in this meta-analysis. The incidence of EBER positivity in these studies ranged from 1.4% to 14.9%. Ten studies were from Asian countries [10,11,15–20,22,24], two from Caucasian countries [14,23], and one from Peru [21]. The HRs with 95% CIs were directly extracted in 9 original studies [11,14,15,18–20,22–24]. Four studies [10,16,17,21] indirectly calculated these values from Kaplan-Meier curves proposed by Tierney et al. [26]. In terms of methodological quality, all included studies scored more than six points on the NOS, indicating a relative high quality.

**Table 1:** Main characteristics of the eligible studies.

First author	Year	Setting	No. of patients (EBV+/EBV-)	EBER cut-off	Method	Median follow-up(months)	Outcome	Data extraction	NOS
Park S	2007	Korea	380(34/346)	20%	ISH	40.5	OS/PFS	Kaplan-Meier curve	7
Morales D	2010	Peru	74(11/63)	NR	ISH	NR	OS	Direct	7
Chang ST	2013	Taiwan	332(15/317)	10%	ISH	14	OS	Direct	7
Sato A	2014	Japan	239(14/225)	30%	ISH	25.2	OS	Direct	7
Ok CY	2014	USA	703(28/675)	10%	ISH	42.1	OS/PFS	Direct	7
Lu CH	2014	Taiwan	89(15/74)	20%	ISH	NR	OS	Kaplan-Meier curve	6
Liang JH	2015	China	232(24/208)	50%	ISH	38	OS	Direct	7
Lu TX	2015	China	250(35/215)	20%	ISH	29.3	OS/PFS	Kaplan-Meier curve	6
Okamoto A	2015	Japan	134(11/123)	20%	ISH	40	OS/PFS	Kaplan-Meier curve	6
Chuang WY	2015	Taiwan	174(10/164)	10%	ISH	120	OS	Direct	8
Hong JY	2015	Korea	571(48/523)	20%	ISH	102.5	OS	Direct	9
Tracy SI	2018	USA	362(16/346)	30%	ISH	59	OS	Direct	8
Hong JY	2017	Korea	571(48/523)	20%	ISH	42.2	OS/PFS	Direct	8

EBV: Epstein–Barr virus; EBER: Epstein–Barr virus-encoded small RNA; ISH: In situ hybridization; NR: Not Reported; OS: overall survival; PFS: progression-free survival; NOS: Newcastle-Ottawa Scale.

### Overall analyses

Data on the HRs for the correlation between EBER positivity and OS were available in all 13 studies [10,11,14–24], while those for PFS were available in 5 studies [10,14,16–18]. The overall estimate demonstrated that EBER positivity in DLBCL was correlated with unfavorable outcome for OS (HR = 2.43, 95% CI: 1.73–3.36, *P*<0.001).

**Fig 2:**
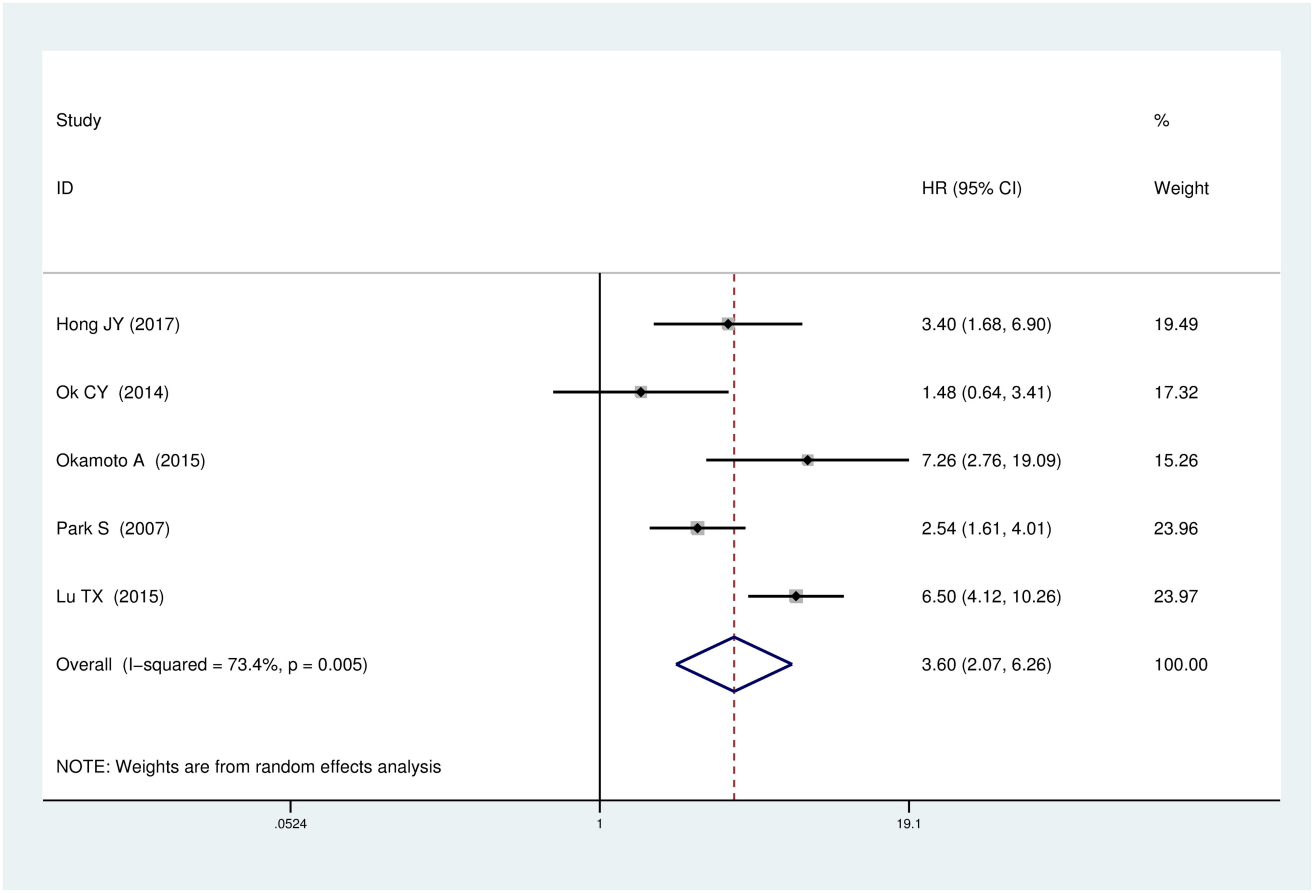
Forest plot of the hazard rations for progression free survival (PFS) between patients with EBER-positive and EBER-negative DLBCL.

The heterogeneity is moderate (I^2^ = 61.1%, *P* = 0.002) among these studies; therefore, a random-effects model was adopted to elevate the pooled HR. The pooled HR on PFS was 3.60 (95% CI, 2.07–6.26, *P*<0.001) as calculated via random-effects model for the existence of a considerable heterogeneity (I^2^ = 73.4%, *P* = 0.005) (Fig 2). This result showed a distinctly increased risk of disease progression in the EBER-positive DLBCL group.

### Subgroup analyses

**Fig 3:**
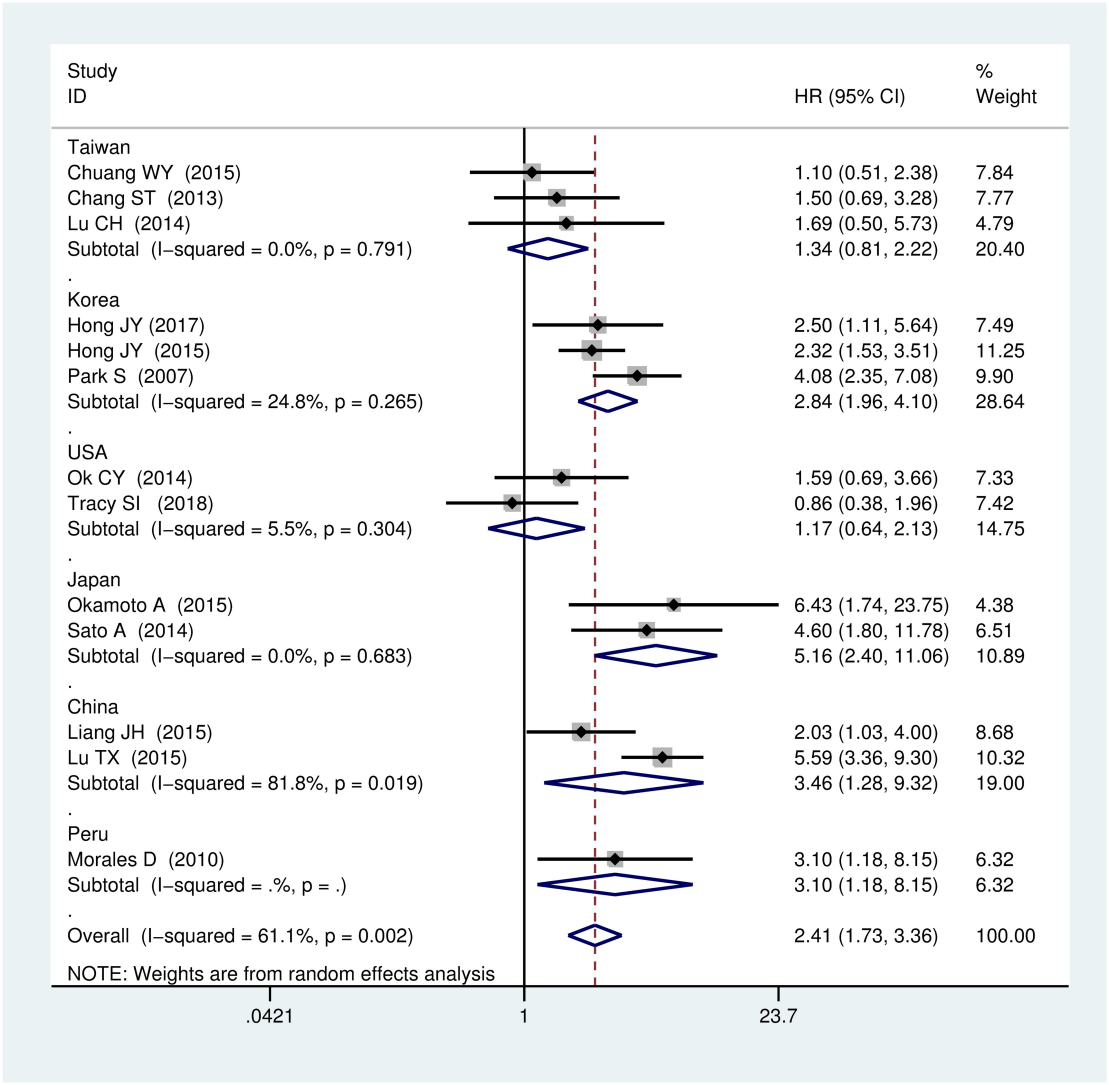
Forest plot of the hazard rations for overall survival (OS). Subgroup analysis was according to the different study setting.

To explain the heterogeneity, we conducted a subgroup analysis by geographical location using a random-effects model. In the analysis of OS, we divided the patients into different geographical groups as follows: Korea, Japan, China, USA, Taiwan, and Peru (Fig 3). Worse prognosis was strongly correlated to EBER positivity in Korea (n = 3 studies, HR = 2.84, 95% CI: 1.96–4.10, I^2^ = 24.8%), Japan (n = 2 studies, HR = 5.16, 95% CI: 2.40–11.06, I^2^ = 0.0%), Peru (n = 1 study, HR = 3.10, 95% CI: 1.18–8.15), and China (n = 2 studies, HR = 3.46, 95% CI: 1.28–9.32, I^2^ = 81.8%). By contrast, there was no significant difference in Taiwan (n = 3 studies, HR = 1.34, 95% CI: 0.81–2.22, I^2^ = 0.0%) and USA (n = 2 studies, HR = 1.17, 95% CI: 0.64–2.13, I^2^ = 5.5%).

### Correlation between EBER positivity and clinical features

**Fig 4:**
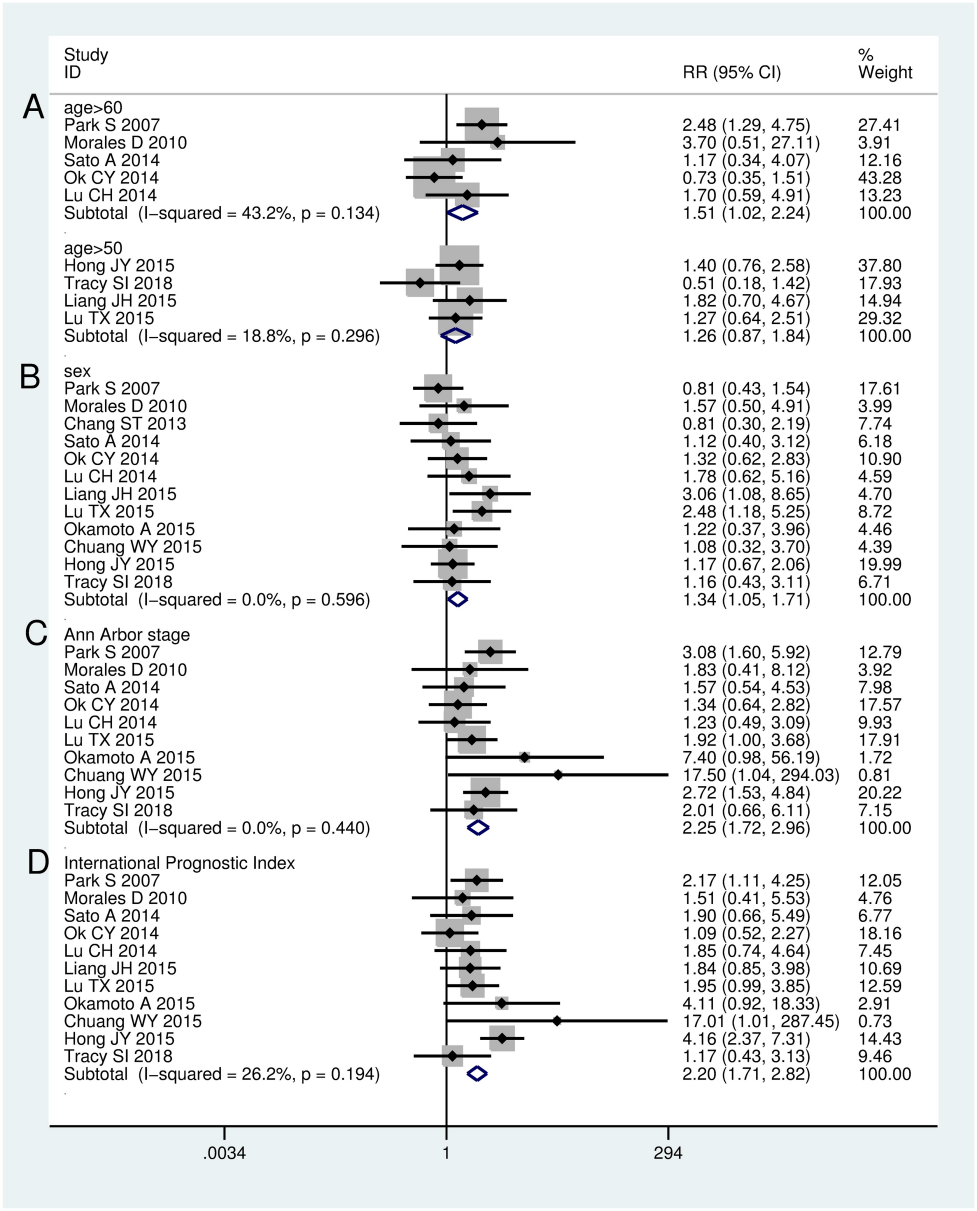
Meta-analysis of the association between EBER positivity and clinical features in DLBCL patients. (A) age (>60 vs <60,>50 vs <50); (B) sex (male vs female); (C) Ann Arbor stage(I +II vs III+IV); (D) International Prognostic Index(1–2 vs 3–4).

**Fig 5:**
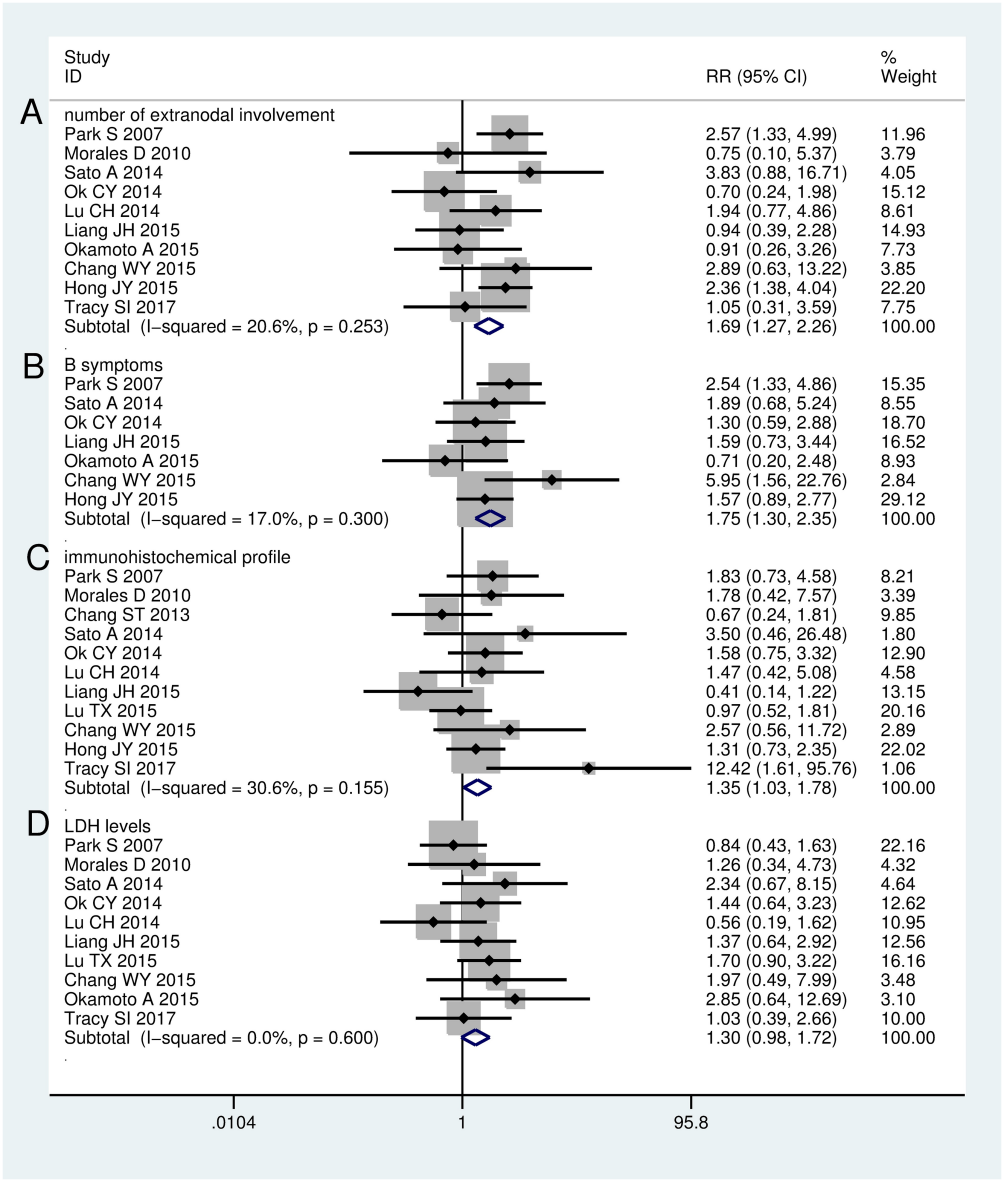
Meta-analysis of the association between EBER positivity and clinical features in DLBCL patients. (A) number of extranodal involvement(>1 vs ≤1); (B) B symptom(presence vs absence); (C) immunohistochemical profile(non-GCB vs GCB); (D) LDH levels(elevated vs normal).

Ten studies had information on the correlation between EBER positivity and age in DLBCL. A meta-analysis of five studies [10,11,14,19,21] showed an association between EBER positivity and age >60 years (RR = 1.51, 95% CI: 1.02–2.24). The heterogeneity across these studies was moderate (I^2^ = 43.2%, *P* = 0.134). However, a meta-analysis of four studies [15,16,20,23] suggested that EBER positivity was not significantly correlated to age >50 years (RR = 1.26, 95% CI: 0.87–1.84). No considerable heterogeneity was noted across these studies (I^2^ = 18.8%, *P* = 0.296) (Fig 4A). An analysis of twelve studies [10,11,14–17,19–24] revealed that EBER positivity was more prevalent in men (RR = 1.34, 95% CI: 1.05–1.71). No heterogeneity was noted across these studies (I^2^ = 0.0%, *P* = 0.596) (Fig 4B). An analysis of ten studies [10,11,14,16,17,19–21,23,24] revealed that EBER positivity was correlated with more advanced disease stage (I+II vs III+IV, RR = 2.25, 95% CI: 1.72–2.96). No heterogeneity was noted across these studies (I^2^ = 0.0%, *P* = 0.440) (Fig 4C). An analysis of eleven studies [10,11,14–17,19–21,23,24] indicated that EBER positivity was associated with higher international IPI score (1–2 vs 3–4, RR = 2.20, 95% CI: 1.71–2.82). The heterogeneity across these studies was moderate (I^2^ = 26.2%, *P* = 0.194) (Fig 4D). An analysis of ten studies [10,11,14,15,17,19–21,23,24] indicated that EBER positivity was associated with more than one extranodal involvement (RR = 1.69, 95% CI: 1.27–2.26). The heterogeneity across these studies was moderate (I^2^ = 20.6%, *P* = 0.253) (Fig 5A). An analysis of seven studies [10,11,14,15,17,20,24] indicated that EBER positivity was associated with the presence of B symptom (RR = 1.75, 95% CI: 1.30–2.35). No apparent heterogeneity was noted across these studies (I^2^ = 17.0%, *P* = 0.300) (Fig 5B). An analysis of eleven studies [10,11,14–16,19–24] indicated that EBER positivity was associated with non-germinal center B-cell (GCB) subtype (RR = 1.35, 95% CI: 1.03–1.78). The heterogeneity across these studies was moderate (I^2^ = 30.6%, *P* = 0.155) (Fig 5C). An analysis of ten studies [10,11,14–17,19,21,23,24] indicated that EBER positivity tended to be associated with elevated LDH levels (RR = 1.30, 95% CI: 0.98–1.72). No heterogeneity was noted across these studies (I^2^ = 0.0%, *P* = 0.600) (Fig 5D).

### Sensitivity analyses and publication bias

To evaluate the stability of results, we conducted a sensitivity analysis by excluding each eligible study. The results showed that no individual study substantially influenced the pooled HR for OS (S1 Fig). Begg’s and Egger’s tests for estimating publication bias for OS in this meta-analysis showed no substantial publication bias (Begg’s test, *P* = 1 (S2 Fig); Egger’s test, *P* = 0.476 (S3 Fig)).

## Discussion

Although many studies demonstrated that EBER positivity indicated worse clinical features and poor prognosis in DLBCL patients, there is still no unified conclusion. Therefore, we conducted the first meta-analysis of published literature to systemically explore the prognostic value of EBV positivity in DLBCL. Our results demonstrated that EBER positivity significantly predicted poor PFS and short OS (increased risk of tumor relapse and death). Moreover, subgroup analysis showed that EBER positivity is related to inferior clinicopathological parameters and aggressive clinical course in DLBCL patients. Thus, EBER positivity should be considered as a valuable prognostic marker and risk-stratifying factor in DLBCL.

Consistent with other findings, our results revealed that patients with EBER-positive DLBCL tended to be men, aged >60 years, who had higher IPI scores, a non-GCB immunophenotype, more than one extranodal site, an advanced disease stage, B symptom, and elevated LDH levels. These clinical features correspond to the invasive clinical course of DLBCL. Subgroup analysis might reveal the reason by which EBER positivity was associated with poor OS and PFS among patients with DLBCL. Several studies strongly suggest that EBV-associated viral proteins play an important role in oncogenesis, including regulation of proliferation, metastasis, immune escape, and cell apoptosis [27–29]. Latent membrane protein 1 (LMP1), an important oncogenic protein encoded by EBV, stimulate proliferation of B cells via activate nuclear factor-kappa B (NFκB) and transcription factor AP-1[30]. LMP1 can also inhibit the apoptosis of tumor cells by inducing the expression of anti-apoptotic genes A20 and Bcl-2 [31]. Epstein-Barr nuclear antigen 1 (EBNA1) can restrict the translation of its own mRNA through a specific region and suppress antigenic peptides combined with major histocompatibility complex 1 (MHC-1) [32], which can cause virus-carrying host cells to evade the identification of cytotoxic T lymphocytes (CTL), thus avoiding the apoptotic action of the immune system. Moreover, EBNA1 can prevent the degradation of lytic enzymes and the activation of CTL [33]. When the normal immune function is compromised, malignant neoplasms develop easier. Collectively, these mechanisms can explain the association between EBER positivity and unfavorable clinicopathological parameters in DLBCL patients. Thus, EBER positivity can be a reliable marker for predicting the prognosis of DLBCL, compensating for the deficiency in the current prognostic scoring system.

However, our meta-analysis has several limitations. First, a certain degree of heterogeneity existed among the studies for the overall meta-analysis. We investigated the potential sources of the heterogeneity using subgroup analysis and found that most of the heterogeneity was derived from the different regions of the studies. In this subgroup analysis, a significant correlation of EBER positivity with OS was noted in Korea, Japan, China, and Peru. By contrast, there was no significant correlation in Taiwan and the USA, which might be related to differences in race and lifestyle. Interestingly, the frequency of EBV-positive DLBCL was higher in Korea (6.7%–8.9%) [10,18,20], Japan (5.9%–8.2%) [11,17], China (10.3%–14%) [15,16], and Peru(14.9%) [19] than in Taiwan (1.7%–5.7%)) [21,22,24] and the USA (4.0%–4.4%) [14,23]. The elderly may develop immunodeficiency-associated lymphoproliferative disorders through senescence of the immune system as part of the normal aging process. This leads to low-grade chronic infections such as EBV infection [14]. Thus, the age distribution and the ageing index (ratio of the number of people aged ≥65 years to the number of those aged ≤15 years) might be related to the different incidence of EBV, resulting in a diverse risk of DLBCL in different geographic regions. In addition, although laboratory tests should not be the major source of variation because we only included studies that evaluated EBER via in-situ hybridization of paraffin-embedded tissue, subtle differences in technical quality cannot be eliminated. EBER detection via in-situ hybridization is considered the gold standard for evaluating EBER positivity, but this method is a semi-quantitative analysis, and, to a certain degree, the reported results depend on the observer [17]. Other unidentified factors may have played a role in the heterogeneity of the current meta-analysis. Second, there is no established standard for the proportion of EBV-positive cells in EBV-positive DLBCL, which is a limitation when evaluating disease prevalence [13]. Some studies reported that the different cut-off values for EBER-positive cells are bound to influence the prevalence of EBV-positive DLBCL [34,35]. As shown in Table 1, the criteria for defining EBV-positive cases varied among the included studies and ranged from 10% to 50%, while it was not defined in one study [19]. Specifically, a study from China adopting the two most commonly used cut-off values of >20% and >50% reported EBV-positive rates of 14% and 10.4%, respectively [16]. However, this study showed that EBER-positive patients harbored inferior prognosis and aggressive clinical course compared with EBER-negative patients irrespective of the cut-off values of EBER positivity. Even so, a unified cut-off value for EBV positivity still need to be established and should either be >20%, >30%, or 100%. Third, some HRs with 95% CI calculated from the survival curves might be less reliable than those directly extracted from the original study. Finally, we have included all available data, but we only found most published studies from Asia, especially the lack of Europe data. This emphasises the need for future studies to test the association of EBV infection with prognosis in DLBCL in the Western countries, generally lower prevalence of EBER-positive DLBCL.

In conclusion, the results of the meta-analysis revealed that EBER positivity in patients with DLBCL is associated with worse clinical course and poor survival. EBER positivity tended to be correlated with male sex, age older than 60 years, high IPI score, more advanced staged, more than one extranodal involvement, the presence of B symptom, non-GCB subtype, and elevated LDH levels. EBER positivity might be a useful predictive indicator of poor outcome and can be used in determining appropriate individualized treatment modality for patients with DLBCL. More high-quality and larger prospective clinical studies with a standard cut-off value for EBV positivity are essential to accurately define the role of EBV in the prognosis of DLBCL.

## Supporting information

S1 File PRISMA checklist. (DOC)

S2 File Search strategy of this study. (DOCX)

S1 Fig Sensitivity analysis for the pooled HRs in OS. (TIF)

S2 Fig Begg’s funnel plots of the prognostic role of EBER positivity in OS. (TIF)

S3 Fig Egger’s linear regression test of the prognostic role of EBER positivity in OS. (TIF)
