# Supplementary material for: Clinical characteristics and prognostic significance of EBER positivity in diffuse large B-cell lymphoma: A meta-analysis
Source: PLoS One. 2018 Jun 19;13(6):e0199398. doi: 10.1371/journal.pone.0199398 (PMC6007832; doi:10.1371/journal.pone.0199398)
Supplement: S2 File — (DOCX) [file pone.0199398.s002.docx]

**Search strategy pubmed:**

| #3 | #1 AND #2 |
| --- | --- |
| #2 | Search ((((("Epstein-Barr Virus"[Title/ Abstract]) or "EB virus"[Title/Abstract]) or "EBV"[Title/Abstract]) or "human herpesvirus 4"[Title/Abstract]) or "HHV 4"[Title/Abstract]) |
| #1 | Search (("diffuse large B cell lymphoma"[Title/Abstract]) or "DLBCL"[Title/ Abstract]) |

**Search strategy web of science:**

| # 3 | #2 AND #1  Refined by: LANGUAGES: (ENGLISH)  Indexes=SCI-EXPANDED, SSCI, CPCI-S, CCR-EXPANDED,  IC Timespan=1950-2018 |
| --- | --- |
| # 2 | TS=("Epstein-Barr Virus") OR TS=("EB virus") OR TS=("EBV") OR TS=("human herpesvirus 4") OR TS=("HHV 4")  Refined by: LANGUAGES: (ENGLISH)  Indexes=SCI-EXPANDED, SSCI, CPCI-S, CCR-EXPANDED,  IC Timespan=1950-2018 |
| # 1 | TS=("DLBCL") OR TS=("diffuse large B cell lymphoma")  Refined by: LANGUAGES: (ENGLISH)  Indexes=SCI-EXPANDED, SSCI, CPCI-S, CCR-EXPANDED,  IC Timespan=1950-2018 |
