# Supplementary figures and images for: Clinical characteristics and prognostic significance of EBER positivity in diffuse large B-cell lymphoma: A meta-analysis
Source: PLoS One. 2018 Jun 19;13(6):e0199398. doi: 10.1371/journal.pone.0199398 (PMC6007832; doi:10.1371/journal.pone.0199398)

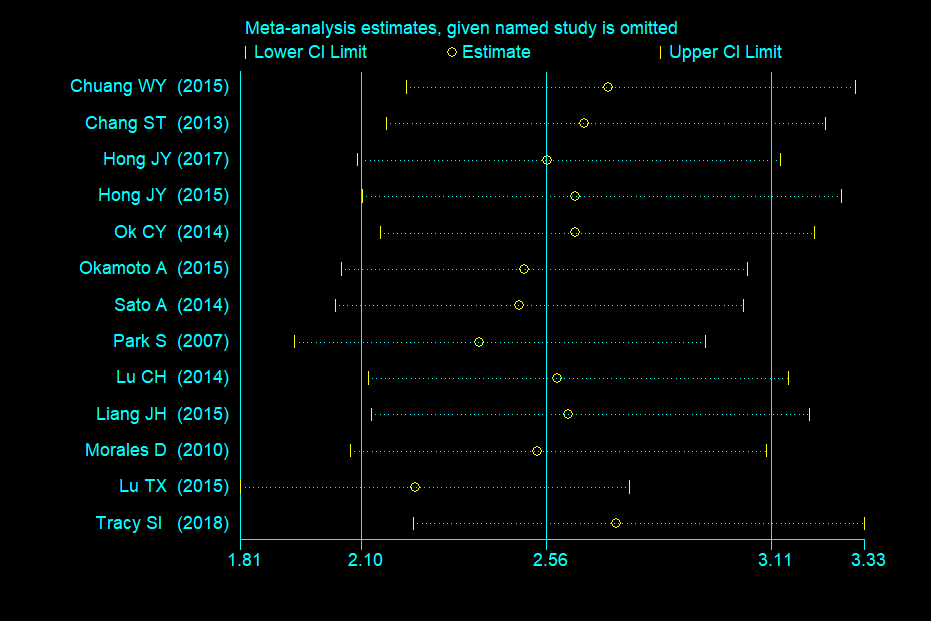

Supplement: S1 Fig — (TIF) [file pone.0199398.s003.tif]

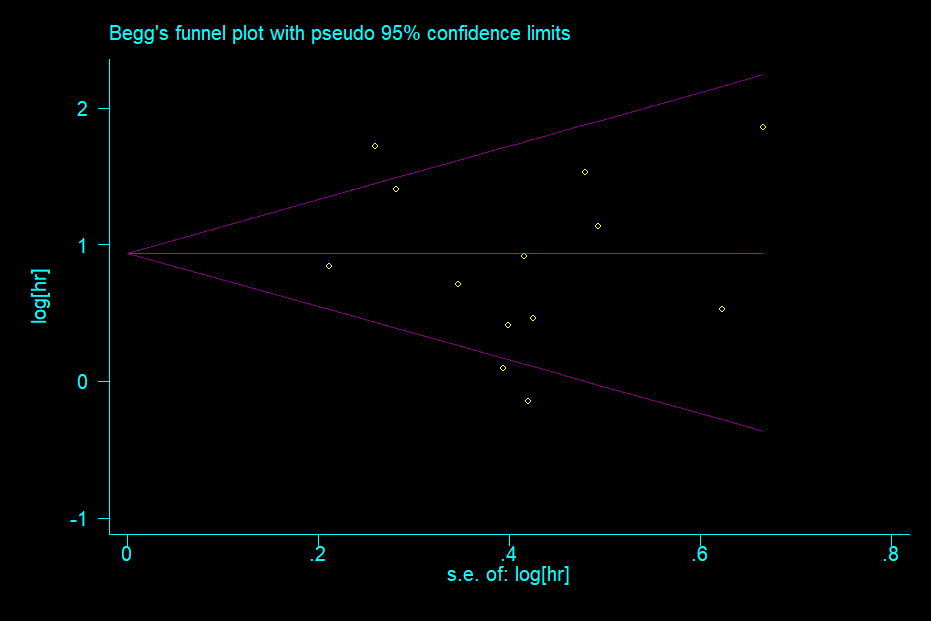

Supplement: S2 Fig — (TIF) [file pone.0199398.s004.tif]

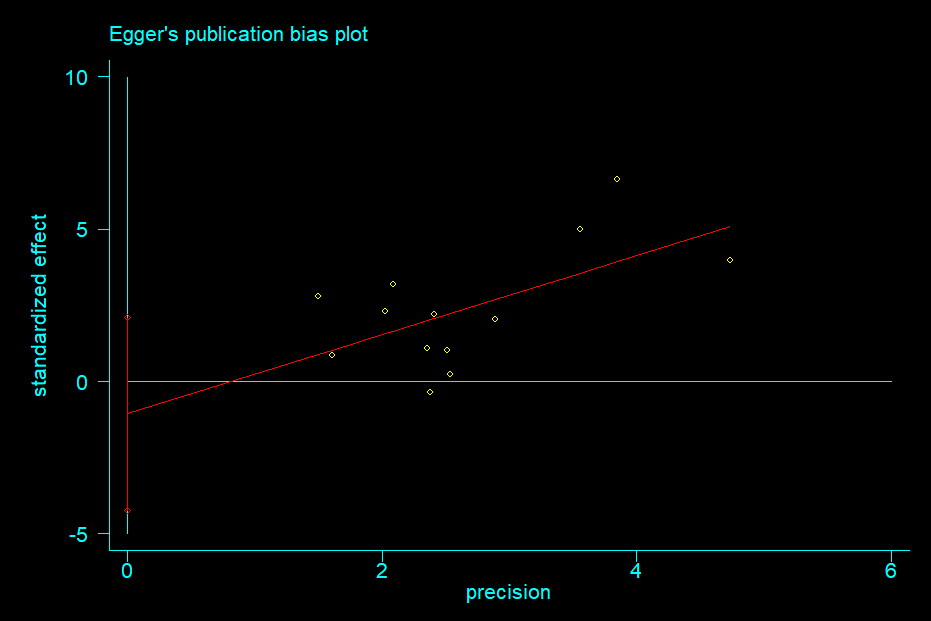

Supplement: S3 Fig — (TIF) [file pone.0199398.s005.tif]
